# On the utility of a compartmental population kinetics model of intestinal epithelial stem cell proliferation and differentiation

**DOI:** 10.1186/s12976-017-0071-8

**Published:** 2017-12-19

**Authors:** Erik R. Barthel

**Affiliations:** Department of Surgery, Section of Pediatric Surgery, University of Chicago Biological Sciences Division, 5841 S Maryland Ave MC 4062, Chicago, IL 60637 USA

## Abstract

**Background:**

The small intestinal epithelium is a dynamic system with specialized cell types. The various cell populations of this tissue are continually renewed and replenished from stem cells that reside in the small intestinal crypt. The cell types and their locations in the crypt and villus are well known, but the details of the kinetics of stem cell division, and precursor cell proliferation and differentiation into mature enterocytes and secretory cells are still being studied. These proliferation and differentiation events have been extensively modeled with a variety of computational approaches in the past.

**Methods:**

A compartmental population kinetics model, incorporating experimentally measured proliferation rates for various intestinal epithelial cell types, is implemented for a previously reported scheme for the intestinal cell dynamics. A sensitivity analysis is performed to determine the effect that varying the model parameters has upon the model outputs, the steady-state cell populations.

**Results:**

The model is unable to reproduce the experimentally known timescale of renewal of the intestinal epithelium if literature values for the proliferation rates of stem cells and transit amplifying cells are employed. Unphysically large rates of proliferation result when these parameters are allowed to vary to reproduce this timescale and the steady-state populations of terminally differentiated intestinal epithelial cells. Sensitivity analysis reveals that the strongest contributor to the steady-state populations is the transit amplifying cell proliferation rate when literature values are used, but that the differentiation rate of transit amplifying cells to secretory progenitor cells dominates when all parameters are allowed to vary.

**Conclusions:**

A compartmental population kinetics model of proliferation and differentiation of cells of the intestinal epithelium can provide a simplifying means of understanding a complicated multistep process. However, when literature values for proliferation rates of the crypt based columnar and transit amplifying cell populations are employed in the model, it cannot reproduce the experimentally known timescale of intestinal epithelial renewal. Nevertheless, it remains a valuable conceptual tool, and its sensitivity analysis provides important clues for which events in the process are the most important in controlling the steady-state populations of specialized intestinal epithelial cells.

**Electronic supplementary material:**

The online version of this article (10.1186/s12976-017-0071-8) contains supplementary material, which is available to authorized users.

## Background

The cell dynamics of the small intestine epithelium is increasingly well studied from both an experimental as well as a theoretical direction. The population and maintenance of its finely-tuned balance of absorptive and secretory cell populations from the intestinal crypt has become an archetypal example of homeostasis regulated by a stem cell niche. It has been demonstrated by the Clevers group that the intestinal stem cell is the crypt based columnar (CBC) cell that resides between Paneth cells at the crypt base and expresses the marker Lgr5 [1]. These stem cells divide both to maintain their own population and remain at the base of the crypt, and to produce proliferative transit amplifying cells that migrate up the crypt [2, 3], and further divide and differentiate into terminally differentiated cell populations of the intestinal epithelium: the absorptive enterocytes; and the secretory goblet cells [4]; enteroendocrine cells [5, 6]; and Paneth cells [7–9]. Another secretory cell, the Tuft cell, has also been described [10]. Each crypt has about 250 cells, and each villus, about 3500 cells [8], although these values vary depending on the position along the small bowel [11]. The signaling mechanisms governing the fate of transit amplifying cells to enterocytes or one of the secretory cell types are complex and under active study, but broadly include the Wnt pathway, which regulates proliferation in the crypt base, and Notch signaling, which determines whether transit amplifying cells and other intermediate cell populations will go down the absorptive or secretory pathways [12].

The complexity of the population dynamics of the intestinal epithelium, combined with the continually evolving amount of experimental data available about the system, has long made it an attractive target for mathematical simulation [13]. Moreover, the 3-dimensional structure of the crypt, and crypt-villus unit in the small intestine, naturally lends itself to models incorporating a spatial component. One significant early approach was that of a stochastic lattice model, early examples of which, while constructed before the definitive identity of the CBC cell as the intestinal stem cell, nevertheless correctly predicted the location of the stem cells as being in close contact with Paneth cells at the bottom of the crypts [14, 15]. More recently, multiscale models have been proposed that incorporate population dynamics, signaling, and the topology of the crypt without the constraint of a lattice [16–18]; these models include a cell-cell surface interaction using intercellular springs obeying Hooke’s Law. A compartmental Monte Carlo model has also been described [19]. This work was able to reproduce the experimentally known localization of Paneth cells to the crypt base without the need for a constraint force to be built into the model. Another approach for a variety of modeling problems is that of agent-based modeling, which has recently been employed to simulate colonic crypts both in normal steady-state conditions and in colon cancer [20]. These authors used the NetLogo platform [21] to construct a model which was calibrated to experimental data for normal crypt dynamics, but which could also be tuned to simulate overproliferation in cancer, as well as the effects of cytotoxic chemotherapy. Perhaps one of the most comprehensive approaches described to date incorporated both mechanical cell-cell interaction and cell interaction with Wnt and Notch signaling from their local environment, and has been used to simulate both intestinal crypts as well as organoids in culture [22, 23].

During the differentiation process in the small intestinal epithelium, it has been suggested that there are common progenitor cells that may differentiate into various subsets of the five terminally differentiated populations. For example, it has been proposed that the goblet cell compartment arises, at least in part, from an “oligomucous” cell type that, while maintaining proliferative ability, nevertheless is committed to goblet cell differentiation [24–26]. More recently, a progenitor cell specific for the Paneth and goblet cell populations, or “intermediate cell” [27–29] has been described. Samuelson and coworkers [30] were able to demonstrate experimentally the presence of such a progenitor population based upon co-immunofluorescence staining for immunofluorescent markers of both goblet and Paneth cells. They summarized their work in a simple but conceptually elegant scheme for how cells in the intestinal epithelium divide and differentiate into the terminally differentiated cell populations, including the newly demonstrated progenitors. A compartmental scheme like this lends itself naturally to a deterministic compartmental model of the cell dynamics. The goal of this work was to apply the simplicity of a first-order, ordinary differential equations compartmental kinetics model to the well-defined scheme for the intestinal epithelium described in [30], constrained by experimentally known timescales for epithelial renewal and proliferation for some of the epithelial cell populations, in order to extract the differentiation rates of those cell types for which no experimental value is yet known. For this reason, our model differs from the previously discussed examples, in that it does not include any information about the topologic layout of the crypt-villus axis. Rather, it is entirely deterministic, and information about movement of cells along that axis is contained only implicitly, in averaged way, in the differential equations describing movement between compartments. It is important to remark that the model described in reference [19] incorporated intermediate cells (goblet/Paneth progenitors, as well as absorptive progenitors). Also, Johnston and coworkers have compared an age-structured model of the colonic crypt with a compartmental model similar in many respects to the ODE approach taken here [31]. In simulating colorectal cancer with a continuous model, these workers found that increased cell renewal can result in tumor growth. However, to our knowledge, the current work is the first to employ a continuous, ODE-type compartmental model for intestinal epithelial proliferation and differentiation with the goal of extracting unknown rates of progenitor proliferation and differentiation.

## Methods

### Modeling approach

**Fig. 1:**
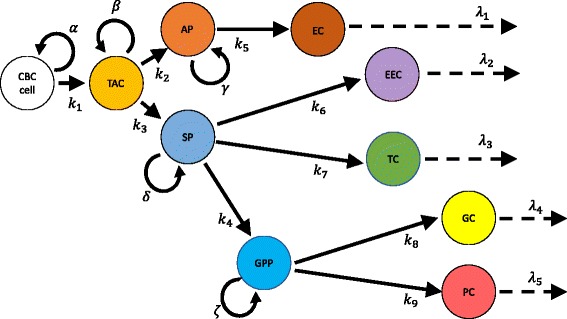
Compartmental scheme for proliferation and differentiation of the small intestinal epithelium, based (with permission) on Figure 7 of ref. 30. Subscripted *k*’s represent differentiation rates; unscripted lowercase Greek letters (*α*, *β*, *γ*, *δ*, *ζ*) indicate proliferation rates; subscripted *λ*’s indicate cell loss rates. Colors match the scheme in Figure 7 of ref. 26. CBC: crypt based columnar cell; TAC: transit amplifying cell; AP: absorptive progenitor; EC: enterocyte; SP: secretory progenitor; EEC: enteroendocrine cell; TC: Tuft cell; GPP: goblet / Paneth progenitor; GC: goblet cell; PC: Paneth cell

We apply a first order, ordinary differential equations (ODE) compartmental model to the scheme published by Samuelson’s group as shown in Fig. 7 of ref. [30]. We have (with permission) reproduced and modified it slightly in Fig. 1. We identify the yellow-colored cell population in their figure as the transit-amplifying cell (TAC) and assign phenomenological rate constants to each differentiation event. This leads to the series of differential equations:

1 $$ \frac{dCBC}{dt}=-\left({k}_1-\alpha \right) CBC $$

2 $$ \frac{dTAC}{dt}=-\left({k}_2+{k}_3-\beta \right) TAC+{k}_1 CBC $$

3 $$ \frac{dAP}{dt}={k}_2 TAC-\left({k}_5-\gamma \right) AP $$

4 $$ \frac{dEC}{dt}={k}_5 AP-{\lambda}_1 EC $$

5 $$ \frac{dSP}{dt}={k}_3 TAC-\left({k}_4+{k}_6+{k}_7-\delta \right) SP $$

6 $$ \frac{dEEC}{dt}={k}_6 SP-{\lambda}_2 EEC $$

7 $$ \frac{dTC}{dt}={k}_7 SP-{\lambda}_3 TC $$

8 $$ \frac{dGPP}{dt}={k}_4 SP-\left({k}_8+{k}_9-\zeta \right) GPP $$

9 $$ \frac{dGC}{dt}={k}_8 GPP-{\lambda}_4 GC $$

10 $$ \frac{dPC}{dt}={k}_9 GPP-{\lambda}_5 PC $$

where the symbols have the meanings described in Tables 1 and 2. We note that for a constant, steady-state population of crypt-based columnar cells, by construction *k*_1_ must equal *α*, or equivalently, $\frac{dCBC}{dt}=0$. It is helpful to define

11 $$ {\displaystyle \begin{array}{l}{\omega}_d\equiv {k}_2+{k}_3-\beta \\ {}{\omega}_e\equiv {k}_5-\gamma \\ {}{\omega}_s\equiv {k}_4+{k}_6+{k}_7-\delta \\ {}{\omega}_m\equiv {k}_8+{k}_9-\zeta \end{array}} $$

**Table 1:** Parameters employed in kinetics model of intestinal stem cell differentiation

Parameter	Meaning	Units
*CBC*	Number of crypt-base columnar cells	None
*TAC*	Number of transit-amplifying cells	None
*AP*	Number of absorptive progenitor cells	None
*EC*	Number of enterocytes	None
*SP*	Number of secretory progenitor cells	None
*EEC*	Number of enteroendocrine cells	None
*TC*	Number of tuft cells	None
*GPP*	Number of goblet / Paneth progenitor cells	None
*GC*	Number of goblet cells	None
*PC*	Number of Paneth cells	None
*k*_1_	Crypt-based columnar cell to transit-amplifying cell differentiation rate	day^-1^
*k*_2_	Transit-amplifying cell to absorptive progenitor differentiation rate	day^-1^
*k*_3_	Transit-amplifying cell to secretory progenitor differentiation rate	day^-1^
*k*_4_	Secretory progenitor to goblet / Paneth progenitor cell differentiation rate	day^-1^
*k*_5_	Absorptive progenitor to enterocyte differentiation rate	day^-1^
*k*_6_	Secretory progenitor to enteroendocrine cell differentiation rate	day^-1^
*k*_7_	Secretory progenitor to tuft cell differentiation rate	day^-1^
*k*_8_	Goblet / Paneth progenitor to goblet cell differentiation rate	day^-1^
*k*_9_	Goblet / Paneth progenitor to Paneth cell differentiation rate	day^-1^
*α*	Crypt-based columnar cell proliferation rate	day^-1^
*β*	Transit-amplifying cell proliferation rate	day^-1^
*γ*	Absorptive progenitor cell proliferation rate	day^-1^
*δ*	Secretory progenitor cell proliferation rate	day^-1^
*ζ*	Goblet / Paneth progenitor cell proliferation rate	day^-1^
*λ*_1_	Loss rate of enterocytes	day^-1^
*λ*_2_	Loss rate of enteroendocrine cells	day^-1^
*λ*_3_	Loss rate of tuft cells	day^-1^
*λ*_4_	Loss rate of goblet cells	day^-1^
*λ*_5_	Loss rate of Paneth cells	day^-1^
*N*	Total number of cells per crypt / villus unit	None
*N*_c_	Total number of crypt cells per crypt / villus unit	None
*N*_v_	Total number of villus cells per crypt / villus unit	None

**Table 2:** Additional derived parameters employed in kinetics model

Parameter	Meaning	Definition	Units
*ω*_*d*_	Total transit-amplifying cell loss rate	*k*_2_ + *k*_3_ - *β*	day^-1^
*ω*_*e*_	Total absorptive progenitor cell loss rate	*k*_5_ - *γ*	day^-1^
*ω*_*s*_	Total secretory progenitor cell loss rate	*k*_4_ + *k*_6_+ *k*_7_- *δ*	day^-1^
*ω*_*m*_	Total goblet / Paneth progenitor cell loss rate	*k*_8_ + *k*_9_ - *ζ*	day^-1^

Equations 1, 2, 3, 4, 5, 6, 7, 8, 9, 10 were then solved manually using the integrating factor method [32] and substitution, giving the following expressions for the populations of the cell types in Fig. 1:

12 $$ CBC={CBC}_0 $$

13 $$ TAC=\frac{k_1}{\omega_d}{CBC}_0\left(1-{e}^{-{\omega}_dt}\right) $$

14 $$ AP=\frac{k_1{k}_2}{\omega_d}{CBC}_0\left[\frac{1}{\omega_e}\left(1-{e}^{-{\omega}_et}\right)+\frac{1}{\omega_e-{\omega}_d}\left({e}^{-{\omega}_et}-{e}^{-{\omega}_dt}\right)\right] $$

15 $$ EC=\frac{k_1{k}_2{k}_5}{\omega_d}{CBC}_0\left[\frac{1}{\omega_e{\lambda}_1}\left(1-{e}^{-{\lambda}_1t}\right)\right.+\frac{1}{\left({\omega}_e-{\omega}_d\right)\left({\lambda}_1-{\omega}_d\right)}\left({e}^{-{\lambda}_1t}-{e}^{-{\omega}_dt}\right)\left.+\frac{1}{\left({\lambda}_1-{\omega}_e\right)}\left(\frac{1}{\omega_e}-\frac{1}{\left({\omega}_e-{\omega}_d\right)}\right)\left({e}^{-{\lambda}_1t}-{e}^{-{\omega}_et}\right)\right] $$

16 $$ SP=\frac{k_1{k}_3}{\omega_d}{CBC}_0\left[\frac{1}{\omega_s}\left(1-{e}^{-{\omega}_st}\right)+\frac{1}{\omega_s-{\omega}_d}\left({e}^{-{\omega}_st}-{e}^{-{\omega}_dt}\right)\right] $$

17 $$ EEC=\frac{k_1{k}_3{k}_6}{\omega_d}{CBC}_0\left[\frac{1}{\omega_s{\lambda}_2}\left(1-{e}^{-{\lambda}_2t}\right)\right.+\frac{1}{\left({\omega}_s-{\omega}_d\right)\left({\lambda}_2-{\omega}_d\right)}\left({e}^{-{\lambda}_2t}-{e}^{-{\omega}_dt}\right)\left.+\frac{1}{\left({\lambda}_2-{\omega}_s\right)}\left(\frac{1}{\omega_s}-\frac{1}{\left({\omega}_s-{\omega}_d\right)}\right)\left({e}^{-{\lambda}_2t}-{e}^{-{\omega}_st}\right)\right] $$

18 $$ TC=\frac{k_1{k}_3{k}_7}{\omega_d}{CBC}_0\left[\frac{1}{\omega_s{\lambda}_3}\left(1-{e}^{-{\lambda}_3t}\right)\right.+\frac{1}{\left({\omega}_s-{\omega}_d\right)\left({\lambda}_3-{\omega}_d\right)}\left({e}^{-{\lambda}_3t}-{e}^{-{\omega}_dt}\right)\left.+\frac{1}{\left({\lambda}_3-{\omega}_s\right)}\left(\frac{1}{\omega_s}-\frac{1}{\left({\omega}_s-{\omega}_d\right)}\right)\left({e}^{-{\lambda}_3t}-{e}^{-{\omega}_st}\right)\right] $$

19 $$ GPP=\frac{k_1{k}_3{k}_4}{\omega_d}{CBC}_0\left[\frac{1}{\omega_m{\omega}_s}\left(1-{e}^{-{\omega}_mt}\right)\right.+\frac{1}{\left({\omega}_s-{\omega}_d\right)\left({\omega}_m-{\omega}_d\right)}\left({e}^{-{\omega}_mt}-{e}^{-{\omega}_dt}\right)\left.+\frac{1}{\left({\omega}_m-{\omega}_s\right)}\left(\frac{1}{\omega_s}-\frac{1}{\left({\omega}_s-{\omega}_d\right)}\right)\left({e}^{-{\omega}_mt}-{e}^{-{\omega}_st}\right)\right] $$

20 $$ GC=\frac{k_1{k}_3{k}_4{k}_8}{\omega_d}{CBC}_0\left[\frac{1}{\omega_m{\omega}_s{\lambda}_4}\left(1-{e}^{-{\lambda}_4t}\right)\right.+\frac{1}{\left({\lambda}_4-{\omega}_d\right)\left({\omega}_s-{\omega}_d\right)\left({\omega}_m-{\omega}_d\right)}\left({e}^{-{\lambda}_4t}-{e}^{-{\omega}_dt}\right)+\left(\frac{1}{\omega_s\left({\omega}_m-{\omega}_s\right)}+\frac{1}{\left({\omega}_s-{\omega}_d\right)\left({\omega}_m-{\omega}_d\right)}\right.\left.-\frac{1}{\left({\omega}_s-{\omega}_d\right)\left({\omega}_m-{\omega}_s\right)}-\frac{1}{\omega_m{\omega}_s}\right)\frac{1}{\left({\lambda}_4-{\omega}_m\right)}\left({e}^{-{\omega}_mt}-{e}^{-{\lambda}_4t}\right)+\frac{1}{\left({\lambda}_4-{\omega}_s\right)}\left(\frac{1}{\left({\omega}_s-{\omega}_d\right)\left({\omega}_m-{\omega}_s\right)}-\frac{1}{\omega_s\left({\omega}_m-{\omega}_s\right)}\right)\left({e}^{-{\omega}_st}-{e}^{-{\lambda}_4t}\right) $$

21 $$ PC=\frac{k_1{k}_3{k}_4{k}_9}{\omega_d}{CBC}_0\left[\frac{1}{\omega_m{\omega}_s{\lambda}_5}\left(1-{e}^{-{\lambda}_5t}\right)\right.+\frac{1}{\left({\lambda}_5-{\omega}_d\right)\left({\omega}_s-{\omega}_d\right)\left({\omega}_m-{\omega}_d\right)}\left({e}^{-{\lambda}_5t}-{e}^{-{\omega}_dt}\right)+\left(\frac{1}{\omega_s\left({\omega}_m-{\omega}_s\right)}+\frac{1}{\left({\omega}_s-{\omega}_d\right)\left({\omega}_m-{\omega}_d\right)}\right.\left.-\frac{1}{\left({\omega}_s-{\omega}_d\right)\left({\omega}_m-{\omega}_s\right)}-\frac{1}{\omega_m{\omega}_s}\right)\frac{1}{\left({\lambda}_5-{\omega}_m\right)}\left({e}^{-{\omega}_mt}-{e}^{-{\lambda}_5t}\right)+\frac{1}{\left({\lambda}_5-{\omega}_s\right)}\left(\frac{1}{\left({\omega}_s-{\omega}_d\right)\left({\omega}_m-{\omega}_s\right)}-\frac{1}{\omega_s\left({\omega}_m-{\omega}_s\right)}\right)\left({e}^{-{\omega}_st}-{e}^{-{\lambda}_5t}\right)\Big] $$

We note the behavior of all cell types at long times, which when combined with known differentiation rates will allow us to fit experimentally measured relative abundances of each type of cell. By taking the limit *t* → ∞ we obtain:

22 $$ {TAC}_{t\to \infty }=\frac{k_1}{\omega_d}{CBC}_0 $$

23 $$ {AP}_{t\to \infty }=\frac{k_1{k}_2}{\omega_d{\omega}_e}{CBC}_0=\frac{k_2}{\omega_e}{TAC}_{t\to \infty } $$

24 $$ {EC}_{t\to \infty }=\frac{k_1{k}_2{k}_5}{\omega_d{\omega}_e{\lambda}_1}{CBC}_0=\frac{k_5}{\lambda_1}{AP}_{t\to \infty } $$

25 $$ {SP}_{t\to \infty }=\frac{k_1{k}_3}{\omega_d{\omega}_s}{CBC}_0=\frac{k_3}{\omega_s}{TAC}_{t\to \infty } $$

26 $$ {EEC}_{t\to \infty }=\frac{k_1{k}_3{k}_6}{\omega_d{\omega}_s{\lambda}_2}{CBC}_0=\frac{k_6}{\lambda_2}{SP}_{t\to \infty } $$

27 $$ {TC}_{t\to \infty }=\frac{k_1{k}_3{k}_7}{\omega_d{\omega}_s{\lambda}_3}{CBC}_0=\frac{k_7}{\lambda_3}{SP}_{t\to \infty } $$

28 $$ {GPP}_{t\to \infty }=\frac{k_1{k}_3{k}_4}{\omega_d{\omega}_m{\omega}_s}{CBC}_0=\frac{k_4}{\omega_m}{SP}_{t\to \infty } $$

29 $$ {GC}_{t\to \infty }=\frac{k_1{k}_3{k}_4{k}_8}{\omega_d{\omega}_m{\omega}_s{\lambda}_4}{CBC}_0=\frac{k_8}{\lambda_4}{GPP}_{t\to \infty } $$

30 $$ {PC}_{t\to \infty }=\frac{k_1{k}_3{k}_4{k}_9}{\omega_d{\omega}_m{\omega}_s{\lambda}_5}{CBC}_0=\frac{k_9}{\lambda_5}{GPP}_{t\to \infty } $$

### Definition of a crypt-villus unit (CVU)

**Fig. 2:**
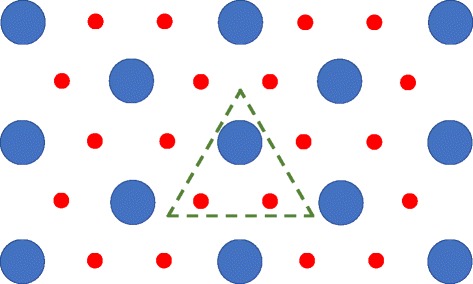
Schematic of hexagonal arrangement of crypts (red) and villi (blue) in mouse ileum. The dashed green triangle represents a unit cell of the 2-dimensional lattice, demonstrating one of several possible arrangements that show that each villus is supplied with cells from, on average, 2 crypts

In order to assign numerical values to known parameters from the model in a consistent fashion, it is necessary to define what comprises a crypt-villus unit or CVU. It has been reported that in the mouse small intestine, six to fourteen crypts surround each villus, with more crypts more proximally [33]. With six crypts per villus, the choice of ileum allows us to simplify the geometry to a hexagonal planar lattice of crypts and villi. This arrangement is shown in Fig. 2. We note that this implies that there are, on average, two complete crypt contributions to each CVU. With this geometric assumption, we can proceed to make quantitative predictions about the contribution of each cell population to the CVU. We also chose to simulate murine ileum given that the goblet / Paneth cell progenitor has been observed there [30].

### Incorporation of experimental data

The relative abundances of the five types of terminally differentiated cell types of the epithelium have been described, as have those of the crypt base columnar cells and rapidly dividing cells. For the purposes of our model, we identify these rapidly dividing cells as transit-amplifying cells. The rate of crypt base columnar cell renewal, average Paneth cell lifetime, and overall renewal rate of the epithelium have also been reported. Also, the steady-state population of transit-amplifying cells is known. These data are collected in Table 3. Of note, our use of the literature parameters 3500 villus cells [8], and a total of 500 crypt cells [9], reported separately, is in line with findings for more distal small bowel in the work of Wright and Irwin, which describes in great detail how the villus and crypt populations vary along the gut axis: these workers found that both unit villus and crypt populations decreased roughly linearly as histologic sections were taken starting at zero, 25%, 50%, 75%, and 100% of the length of the small bowel [11]. Since the number of crypt based columnar cells is fixed in the simulation, this does not contribute a constraint from experimental data. Therefore, we also attempted to constrain the enterocyte population at 5 days to be 95% of its steady state value to reflect the roughly 5-day renewal timescale of the intestinal epithelium [34]. This results in nineteen unknowns (nine differentiation rate constants *k*_*i*_, five progenitor cell proliferation rates *α*, *β*, *γ*, *δ* and *ζ*, and five loss rates *λ*_*i*_) with seven known quantities (the number per crypt villus unit, at long times, of the five terminally differentiated cell types, transit amplifying cells, and the total number of cells at 5 days). We first note the constraint *k*_1_ = *α*, which is necessary for a steady state population of crypt based columnar cells. It has been reported to be 1 day^-1^ [1]. To further reduce the number of free parameters we can also make the assumption that the loss rates of all cells of the secretory lineage are equal, i.e. *λ*_2_ = *λ*_3_ = *λ*_4_, except for the loss rate of Paneth cells, *λ*_5_, which is known to be 1/21 day^-1^ [33]. We also assume that all downstream progenitors divide at the rate reported for the transit amplifying cell, 1.75 ± 0.25 day^-1^ [35] (*β* = *γ* = *δ* = *ζ*= 1.75 day^-1^). We can constrain additional parameters of the model by employing the known abundances of differentiated intestinal epithelial cells at equilibrium and substituting them into eqs. (22)–(30). Whenever there is a branching event in Figure 1 (e.g., TAC to AP or SP), one of the rate constants of the branching can be defined in terms of the other. By convention, in such cases we choose to express the higher subscripted rate in terms of the lower to obtain

31 $$ {k}_3={k}_2\frac{\omega_s}{\omega_e}\frac{SP_{t\to \infty }}{AP_{t\to \infty }} $$

32 $$ {k}_7={k}_6\frac{\lambda_3}{\lambda_2}\frac{TC_{t\to \infty }}{EEC_{t\to \infty }} $$

33 $$ {k}_9={k}_8\frac{\lambda_5}{\lambda_4}\frac{PC_{t\to \infty }}{GC_{t\to \infty }} $$

**Table 3:** Literature values for known parameters and uncertainties (if known) in kinetics model

Parameter	Calculated Value and Units	References
*N*_c_	(250 cells per crypt) x (2 crypts per villus) = 500 crypt cells per crypt/villus unit (see text for details of calculation)	Crosnier [8], Stappenbeck [9]
*N*_v_	3500 villus cells per crypt/villus unit	Crosnier [8]
*N*	4000 total cells per crypt/villus unit (*N* = *N*_*c*_+*N*_*v*_)	––––––––––––––––
*k*_1_ = *α*	1 day^-1^	Barker [1]
*β*	1.75 ± 0.25 day^-1^	Barker [35]
*λ*_5_	(1/21 days) = 0.047 day^-1^	Roth [33]
*CBC*_0_	(5 ± 1 cells per crypt) x (2 crypts per villus) = 10 ± 2 CBC per crypt/villus unit	Marshman [2]
*EEC*_*t* → ∞_	(0.01) x *N* = 40 per crypt/villus unit	Sternini [6], Gunawardene [5]
*TC*_*t* → ∞_	(0.004) x *N* = 16 per crypt/villus unit	Gerbe [10]
*GC*_*t* → ∞_	(0.085 ± 0.015) x *N* = 340 ± 60 per crypt/villus unit	Gregorieff [4]
*PC*_*t* → ∞_	40 ± 10 cells per crypt x (2 crypts per villus) = 80 ± 20 PC per crypt/villus unit	Spradling [7]
*TAC*_*t* → ∞_	155 ± 5 cells per crypt x (2 crypts per villus) = 310 ± 10 TAC per crypt/villus unit	Potten [3]
*EC*_*t* → ∞_	2844 ± 72 (baseline scenario, see text for details of calculation)	––––––––––––––––
*EC*_*t* → ∞_	2848 ± 72 (fast scenario, see text for details of calculation)	––––––––––––––––

The result is seven independent parameters for seven known quantities, allowing for a unique solution.

### Estimates for unknown abundances

Although Samuelson’s group observed goblet / Paneth cell progenitors (GPP), they did not report an abundance for this cell type [30]. Moreover, the absorptive and secretory progenitors (AP and SP) have not been directly observed to date. Therefore, we must estimate the abundances of these three progenitor populations. To accomplish this, we first assume that the abundance of GPP is approximately 1 ± 0.5% based on the figure from the Samuelson paper. We then assume that each progenitor population is proportional to the total daughter populations to which it gives rise. For the GPP this gives

34 $$ {f}_{GPP}=\phi \frac{GC_{t\to \infty }+{PC}_{t\to \infty }}{N} $$

We then solve for *ϕ*, which is found to be 0.0952 with the appropriate values from Table 3. In other words, there are slightly fewer than one order of magnitude fewer goblet / Paneth progenitors than the steady-state sum of their progeny, goblet cells and Paneth cells. We finally assume that this constant of proportionality holds for the other progenitors to obtain

35 $$ {GPP}_{t\to \infty }={f}_{GPP}N=\phi \left({GC}_{t\to \infty }+{PC}_{t\to \infty}\right) $$

36 $$ {AP}_{t\to \infty }={f}_{AP}N=\phi EC $$

37 $$ {SP}_{t\to \infty }={f}_{SP}N=\phi \left({GPP}_{t\to \infty }+{EEC}_{t\to \infty }+{TC}_{t\to \infty }+{GC}_{t\to \infty }+{PC}_{t\to \infty}\right)=\phi \left[{EEC}_{t\to \infty }+{TC}_{t\to \infty }+\left(1+\phi \right)\left({GC}_{t\to \infty }+{PC}_{t\to \infty}\right)\right] $$

This allows calculation of the number of enterocytes at long times as well:

38 $$ {EC}_{t\to \infty }=N-\left({CBC}_0+{TAC}_{t\to \infty }+{AP}_{t\to \infty }+{SP}_{t\to \infty }+{GPP}_{t\to \infty}\right.\left.+{EEC}_{t\to \infty }+{TC}_{t\to \infty }+{GC}_{t\to \infty }+{PC}_{t\to \infty}\right)=\frac{1}{1+\phi}\left[N-\left({CBC}_0+{TAC}_{t\to \infty }+{SP}_{t\to \infty }+{GPP}_{t\to \infty}\right.\right.\left.\left.+{EEC}_{t\to \infty }+{TC}_{t\to \infty }+{GC}_{t\to \infty }+{PC}_{t\to \infty}\right)\right] $$

### Fitting procedure

A Microsoft Excel spreadsheet was constructed to contain the rate constants in eqs. (1)–(11) and the time evolution of all cell populations in eqs. (13)–(21). The calculated time infinity values of the cell populations in eqs. (22)–(30) were fit to the experimental values by varying the rate constants, and the sums of the squares of the residuals were minimized with the Microsoft Excel Solver plug-in. Two scenarios were performed, a baseline scenario (“Additional file 1”), and a fast scenario (“Additional file 2”). For the baseline scenario, experimentally known rate constants from the model were constrained. However, there was no constraint on the amount of time necessary for the enterocyte population to reach 95% of its time infinity value, as this constraint could not be satisfied with the literature values for some of the rate constants in the model, as discussed further in the Results and Discussion sections below. For the fast scenario, all the parameters were allowed to vary (except those which were constrained, as in equations (31)–(33), and the enterocyte population was constrained to reach 95% of its time infinity value at 5 days. Of note, the steady-state enterocyte population *EC*_*t* → ∞_ is constrained by eq. 24 as well, so its value for the fast scenario was 2848. The total number of cells was checked in both fits for consistency to be 4000 per crypt-villus unit.

### Parameter error estimation

Microsoft Excel Solver does not provide the Hessian or curvature matrix after it performs an optimization. To estimate the variance of each fitted parameter, the final fitted values of each were varied by ± 10^-6^ and the increase in the sum of the squares of the residuals was fit to a quadratic equation. The coefficient of the quadratic term is then the reciprocal of twice the variance. The standard deviation of each parameter was then estimated as the square root of the variance obtained. For the constrained parameters, the relative error was estimated as the quadratic sum of the relative errors [36] of the rate constants in the right-hand side of each expression in Eqs. (31)–(33). These calculations were performed in “Additional file 3”. For experimentally known parameters, the literature value of the error was assumed to be the standard deviation. As an example, the error in *k*_7_ was calculated as:

39 $$ {\sigma}_{k_7}={k}_7\sqrt{{\left(\frac{\sigma_{k_6}}{k_6}\right)}^2+{\left(\frac{\sigma_{\lambda_3}}{\lambda_3}\right)}^2+{\left(\frac{\sigma_{\lambda_2}}{\lambda_2}\right)}^2+{\left(\frac{\sigma_{TC}}{TC}\right)}^2+{\left(\frac{\sigma_{EEC}}{EEC}\right)}^2} $$

The subscript *t* → ∞ was dropped from the cell populations *TC* and *EEC* for clarity. For the CBC cell cycling rate *k*_1_ reported in [1], no error estimate was given in the reference, so it was assumed to be 0.25 day^-1^ or 25%. For enterocytes, the error was calculated as the quadratic sum of the errors for the cell populations in the sum given by eq.38.

### Sensitivity analysis

To check the effect of varying the rate constants (model inputs) on the time-infinity values of the cell populations (model outputs), a sensitivity analysis was performed. The approach used is the same as that employed by the author and others in a previous work [37], which relies heavily on the work of Atherton et al. [38]. Briefly, a derivative matrix ***S*** is constructed where the row indices are the outputs (cell populations) and the column indices are differentiation with respect to the inputs (rate constants). If the cell populations are denoted by *P*_*i*_, and the rate constants by *r*_*j*_, then matrix elements of ***S*** are:

40 $$ {S}_{ij}=\frac{\partial {P}_i}{\partial {r}_j} $$

These partial derivatives can be found in “Additional file 4”. The sensitivity of the outputs to the inputs is then tabulated in a variance matrix ***V***, with matrix elements:

41 $$ {V}_{ij}={S_{ij}}^2{\sigma}_{r_j}^2 $$

***S*** and ***V*** for both the baseline and fast scenarios were calculated in “Additional file 5”, and are displayed as Tables S1-S4 in “Additional file 6”. Note that the elements of ***V*** are dimensionless. The *V*_*ij*_ can then be rank-ordered for a given output, allowing the identification of those inputs among the *r*_*j*_ that have the greatest effect on it when varied in the model.

## Results

### Rates of differentiation events in the intestinal epithelium

**Fig. 3:**
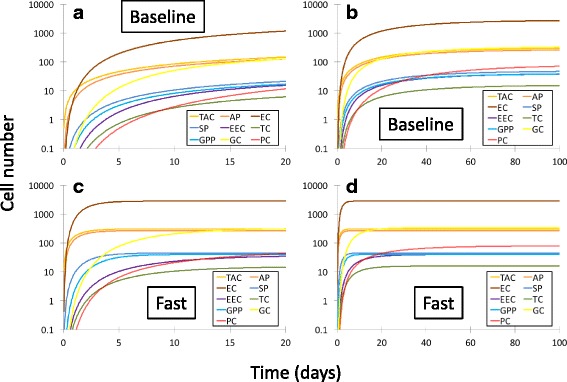
Time dependence of cell populations described by the compartmental population kinetics model in Fig. 1. Colors match those of Fig. 1. The left panels (**a**, **c**) show the results for the baseline and fast scenarios over the first 20 days, respectively; the right panels (**b**, **d**) show the same results over a longer timescale of 100 days. The y axes are displayed in a semilogarithmic format to better display separation between the different cell populations

Figure 1 shows a schematic of the differentiation network from intestinal crypt based columnar cell (CBC) to the five differentiated cell types of the intestinal epithelium, adapted from Samuelson’s recent paper [30]. As described in detail in the Methods section and as shown in the figure, we assigned a rate constant *k*_i_ for each differentiation event, a loss rate *λ*_*i*_ for each terminally differentiated cell type, and proliferation rates for each progenitor population indicated by *α*, *β*, *γ*, *δ* and *ζ*. The results of fitting the known populations of each cell type, with estimates for the AP, SP and GPP populations (eqs. (35)–(37)), are shown in Table 4. The fitted rate constants in Table 4 are those which could be independently varied. We refer to these results as the “baseline scenario.” Table 5 displays the results of rate constants in the baseline scenario that are constrained in terms of the fitted parameters in Table 4. Figure 3 shows plots of all cell populations, described analytically by eqs. (12)–(21), as a function of time for both the baseline and fast scenarios. All populations display a monotonic increase over time. Overall, in both baseline and fast cases, the enterocyte population rises the fastest, followed by the various progenitors, and lastly by the differentiated secretory lineages.

**Table 4:** Estimated values of independent parameters in the baseline scenario

Parameter	Fit value (day^-1^)	Fit uncertainty (day^-1^)
*k*_2_	1.714495	0.000008
*k*_4_	1.67958	0.00005
*k*_5_	3.7112	0.0009
*k*_6_	0.3565	0.0002
*k*_8_	3.712	0.008
*λ*_1_	0.35364	0.00007
*λ*_2_	0.43673	0.00005

**Table 5:** Values of constrained parameters in the baseline scenario

Parameter	Equivalent expression or constraint	Value (day^-1^)	Uncertainty (day^-1^)
*k*_3_	${k}_3={k}_2\frac{\omega_s}{\omega_e}\frac{SP_{t\to \infty }}{AP_{t\to \infty }}$	0.068	0.06
*k*_7_	${k}_7={k}_6\frac{\lambda_3}{\lambda_2}\frac{TC_{t\to \infty }}{EEC_{t\to \infty }}$	0.14	0.10
*k*_9_	${k}_9={k}_8\frac{\lambda_5}{\lambda_4}\frac{PC_{t\to \infty }}{GC_{t\to \infty }}$	0.095	0.045
*β*	Not applicable (literature value)	1.75	0.25
*γ*	*β* = *γ* = *δ* = *ζ*	1.75	0.25
*δ*	*β* = *γ* = *δ* = *ζ*	1.75	0.25
*ζ*	*β* = *γ* = *δ* = *ζ*	1.75	0.25
*λ*_3_	*λ*_2_ = *λ*_3_ = *λ*_4_	0.43673	0.00005
*λ*_4_	*λ*_2_ = *λ*_3_ = *λ*_4_	0.43673	0.00005

### Literature values for crypt based columnar cell proliferation cannot reproduce the observed rate of intestinal epithelial cell renewal

In our fitting procedure, we also made use of the experimental fact that the intestinal epithelium self-renews approximately every 5 days [34]. To do this, we attempted to require that the population of enterocytes reached 95% of its long-time value by *t* = 5 days. However, we found that to accomplish this the progenitor rates downstream of the experimentally fixed value for CBC cell had to be unphysically large, on the order of 120 per day, or five per hour, which greatly exceeds the known rate of cycling of the CBC cell [1]. To further explore this phenomenon, we chose to allow all parameters of the model to vary freely, even those that are known experimentally, to reproduce the correct enterocyte renewal timescale whilst simultaneously preserving the correct long time limit values for each differentiated cell population. The results of this fit are shown in Tables 6 and 7, and we refer to these results as the “fast scenario.”

**Table 6:** Estimated values of independent parameters in the fast scenario

Parameter	Fit value (day^-1^)	Fit uncertainty (day^-1^)
*k*_1_	24.025	0.006
*k*_2_	6.4369	0.0002
*k*_4_	0.798	0.001
*k*_5_	14.875	0.004
*k*_6_	0.1050	0.0002
*k*_8_	1.326	0.004
*β*	5.7868	0.0002
*γ*	7.512	0.002
*δ*	0.096	0.001
*ζ*	0.523	0.002
*λ*_1_	1.4154	0.0004
*λ*_2_	0.1182	0.0002
*λ*_3_	0.148	0.004
*λ*_4_	0.1560	0.0004
*λ*_5_	0.0479	0.0009

**Table 7:** Values of constrained parameters in the fast scenario

Parameter	Equivalent expression or constraint	Value (day^-1^)	Uncertainty (day^-1^)
*k*_3_	${k}_3={k}_2\frac{\omega_s}{\omega_e}\frac{SP_{t\to \infty }}{AP_{t\to \infty }}$	0.125	0.108
*k*_7_	${k}_7={k}_6\frac{\lambda_3}{\lambda_2}\frac{TC_{t\to \infty }}{EEC_{t\to \infty }}$	0.052	0.037
*k*_9_	${k}_9={k}_8\frac{\lambda_5}{\lambda_4}\frac{PC_{t\to \infty }}{GC_{t\to \infty }}$	0.096	0.041

### Summary of sensitivity analysis

As described in the methods, for both the baseline and fast scenarios, a variance matrix was computed to determine the contribution of each input (rate constant) to the sensitivity of each output (time infinity cell populations). The effect of the *j*th input is compared to that of the others for a given cell population by rank order of the values in that row. Table S2 shows the variance matrix for the baseline scenario. In this case, the variance matrix elements are dominated for all time-infinity cell populations by *β*, the transit amplifying cell proliferation rate, followed by *k*_3_, the transit amplifying cell to secretory progenitor cell differentiation rate. For transit amplifying cells, the next most dominant parameter is *k*_1_, the crypt-based columnar cell renewal rate. For the three progenitor populations (absorptive progenitors, secretory progenitors, and Goblet / Paneth cell progenitors), the behavior is somewhat different. The absorptive progenitor population is, like the transit amplifying cell, most sensitive to *β*, *k*_3_, and *k*_1_, but then to *γ* (absorptive progenitor proliferation rate). For the secretory and Goblet / Paneth progenitors, *β* and *k*_3_ are again dominant, but are followed by *δ*, the secretory progenitor proliferation rate. Next, the differentiated secretory lineages (enteroendocrine, tuft, goblet, and Paneth cells), are still most sensitive to *β* and *k*_3_, but the next most important contributions to their error vary from *k*_1_ and *k*_7_ (secretory progenitor to tuft cell differentiation rate) for tuft cells; *δ*, *k*_1_ and *k*_7_ for enteroendocrine cells; and *δ*, *k*_1_, *k*_7_ and *k*_9_ (goblet / Paneth cell progenitor to Paneth cell differentiation rate) for goblet and Paneth cells, with slight differences in their order. Lastly, the enterocyte population behaves like the absorptive progenitor population: it is also most sensitive to *β*, *k*_3_, and *k*_1_, and then to *γ*.

Similarly, Table S4 shows the sensitivity analysis for the fast scenario. The transit amplifying cell population is heavily dominated in the fast scenario by *k*_3_. The progenitor cells also are all dominated by the *k*_3_ contribution, but after this the details change from the baseline scenario. The absorptive progenitor population is relatively insensitive to any parameter other than *k*_3_, but after *k*_3_, the goblet / Paneth progenitor population is roughly equally sensitive to *k*_9_ (goblet / Paneth progenitor to Paneth cell differentiation rate) and *k*_7_. The secretory progenitor population, after *k*_3_, is next most sensitive to *k*_7_ (secretory progenitor to tuft cell differentiation rate). The secretory lineages are roughly split into the enteroendocrine and tuft cell populations, most dependent on *k*_3_ followed by *k*_7_, and the goblet and Paneth cell populations, which are most sensitive to *k*_3_ followed by *k*_9_. Unlike the case of the baseline scenario, in the fast scenario the enterocyte population, while dominated by *k*_3_, is roughly equally sensitive to *k*_1_, *k*_2_, *k*_5_, *γ*, and *λ*_1_, with variance matrix elements of order unity.

## Discussion

A scheme for stepwise proliferation and differentiation for stem cells in the intestinal epithelium, as described in [30], is an extremely useful tool for a conceptual understanding of this complex process. It also allows for researchers to focus on a specific cell population and how it communicates to others in the scheme, and even populations not included, such as the mesenchyme. From a modeling standpoint, such a scheme is by definition compartmental in nature, with well-defined compartments of cells dividing and changing into cells of downstream compartments. This lends itself well to a first-order ODE treatment of the cell division and differentiation dynamics, which in turn allows for a closed-form solution of the time evolution of all the compartments. Such a solution is easily implemented in a spreadsheet or programming languages, and the sensitivity analysis of the model can be done simply as well.

However, the results reported here reveal limitations in the population kinetics approach to modeling the intestinal epithelium. Specifically, in order to reproduce the rapid, 5-day renewal time of the murine intestinal epithelium [34], keeping constant the literature value of 1 per day for the CBC cell, the proliferation rates for the progenitor compartments would have had to be as high as 120 per day (data not shown). Instead, in the fast scenario all nonconstrained rate constants were allowed to vary in order to satisfy the renewal timescale of 5 days, but this still resulted in unphysically large fitted rate constants in the model at the proliferative, “upstream” end of the dynamics. These fitted rate constants differed substantially from values known from the literature: the crypt based columnar cell cycling time *k*_1_ had to be as high as once per hour, 24 times faster than the experimental value. Similarly, the transit amplifying cell proliferation rate *β* was nearly 6 per day, about 3 to 4 times faster than the observed value, in the fast scenario. Interestingly, the Paneth cell loss rate *λ*_5_ did not change much from its literature value of about once every three weeks.

Given the increase seen in *β* in the fast scenario required to reproduce the rapid renewal timescale of the epithelium, it was hypothesized that the absorptive precursor may divide more rapidly than the experimentally known value of 1.75 per day for the transit amplifying cell. Therefore, an attempt was made to fit the observed renewal timescale with *α*, *β* and *λ*_5_ set to their literature values and the other constraints the same as in the baseline scenario, except that the precursor proliferation rates *γ*, *δ* and *ζ* were allowed to vary. However, even this approach did not allow for a fit to the data (not shown). In fact, in the baseline scenario, the total population of cells does not reach 95% of its final value until *t* = 96.8 days. Of note, Fletcher’s group found that depending on assumptions made about crypt geometry, the timescale for a crypt to be populated by progeny from a single stem cell (clonality) is about 71 days using a multiscale model [18]. The similarity of this timescale and our result suggests that the approach taken here of ODEs with a continuous approximation may be better applied to the establishment of clonality of a crypt, rather than the repopulation of the entire crypt. Nevertheless, taken together, all our results make clear that although a compartmental model is a powerful conceptual tool, the first-order ODE approach cannot provide a complete mathematical explanation of the proliferation and differentiation dynamics of the intestinal epithelium system.

The sensitivity analysis for the baseline scenario showed a dominant effect for *β*, the transit-amplifying cell proliferation rate, on all the time-infinity cell populations. This was followed next by *k*_3_, the differentiation rate for transit-amplifying cells to the secretory progenitor population. In contrast, *k*_3_ dominated for the fast scenario, and *β* had almost no effect. This suggests that in the baseline scenario, the deterministic ODE approach overall would have required more rapid proliferation in the TAC compartment, and perhaps also the progenitor compartments, to reproduce the observed rapid intestinal epithelial renewal timescale. This would be required to compensate for the relatively low, once per day cycling time of the CBC cells. This resulted, in the fast scenario, in a much greater *k*_1_ and *β* than are experimentally observed, in order to completely populate the enterocyte compartment by 5 days. The variance matrix for the fast scenario (Table S4) shows that the sensitivity of the model to *k*_1_ and *β* is very similar for all cell populations, suggesting that *β* could be made faster at the expense of a slower *k*_1_ with little change in the ability of the model to fit the time infinity values of each cell compartment. However, since both *k*_1_ and *β* are known experimentally, this suggests either that the deterministic ODE approach does not capture the dynamics of the intestinal epithelium, or that the proliferation rates of the progenitor compartments, assumed in the baseline scenario to all be equal to *β* to minimize the number of unknown parameters in the fit, are in fact much greater. In fact, when allowed to vary freely in the fast scenario, it is notable that *γ*, the absorptive progenitor proliferation rate, increased to 7.5 per day, about 4 times greater than its assigned value of 1.75 per day in the baseline scenario.

There are a number of possible explanations for the failure of the compartmental model presented here to reproduce the known 5-day renewal timescale of the intestinal epithelium. The crypt-villus axis is heavily regulated by Wnt and Bmp signaling pathways. It has been suggested in other modeling work [22, 23] that a gradient of Wnt, with higher concentrations in the crypt base, can keep cells close to the crypt base in a stem-like state or de-differentiate them. There may be an antagonistic effect of a Bmp gradient extending in the opposite direction, highest at the villus tip and lowest in the crypt [8]. We speculate that, from the standpoint of a compartmental, deterministic ODE model, this could increase the effective number of stem cells, averaged over time, which would have the effect of increasing effective upstream proliferation rates in the specific model described here. This would occur in a way which cannot be captured in a first-order ODE model: both because of this effect, and due to random variation of the cell cycle length [18], as well as circadian variation of the villus cell population [11, 39], it is certain that using a single, averaged rate constant for proliferation and differentiation of a single CBC cell compartment will inevitably fail to capture some of the details of the dynamics. Another possibility is cell-cell signaling: Wnt, Notch, or other paracrine factors not yet identified, either from other cells in the crypt or from the mesenchyme, could cause the CBC cell proliferation to increase with cell proximity, causing the first-order approximation of the model to break down. For example, if, as a result of mediation by one of these factors, the rate of CBC cell proliferation were proportional at times to CBC^2^ in eq. 1, there would be a second order term not accounted for in the model as written. These types of effects would lead to nonlinear and time-dependent terms in the differential equations of the model which are not captured in its present form. Lastly, it is possible that other putative stem cell populations, such as the +4 cell [35] could contribute to the proliferation dynamics, resulting effectively in a more rapid proliferative compartment that is not taken into account by the model reported here.

## Conclusions

A compartmental scheme of intestinal epithelial proliferation and differentiation into terminally differentiated cell populations allows for a simple but powerful conceptual framework for understanding the dynamics of this complex system. Such a scheme can be naturally translated to a deterministic, first-order ordinary differential equations (ODE) model of these dynamics. In the model constructed here, we were able to extract a general picture of the relative timescales involved in the dynamics, but could not reproduce the experimentally-known rapid, 5-day timescale of epithelial renewal. To do this required discarding experimentally known cycling times of cell populations in the model. Because of this, the fit values for proliferation and differentiation of the absorptive progenitior, secretory progenitor, and intermediate cell (goblet / Paneth progenitor) populations are unlikely to reflect the true *in vivo* rates for these processes. Despite this, the sensitivity analysis demonstrated some clear trends in how the terminally differentiated cells are influenced by the upstream behavior of their progenitors in the setting of the scheme modeled here. The dynamics of the intestinal epithelium may elude a quantitative description by a compartmental kinetics model, but the conceptual framework remains valuable as a means of understanding its complexity.

## Additional files

Additional file 1: Baseline scenario. (XLSX 28944 kb)

Additional file 2: Fast scenario. (XLSX 50387 kb)

Additional file 3: Uncertainties compiled. (XLSX 45 kb)

Additional file 4: Supplemental Partial Derivatives (DOCX 23 kb)

Additional file 5: Sensitivity Analsysis. (XLSX 26 kb)

Additional file 6: Supplemental Tables. (DOCX 35 kb)

## References

[1] Barker N, van Es JH, Kuipers J, Kujala P, van den Born M, Cozijnsen M, Haegebarth A, Korving J, Begthel H (2007). Peters PJ et al: Identification of stem cells in small intestine and colon by marker gene Lgr5. Nature.

[2] Marshman E, Booth C, Potten CS (2002). The intestinal epithelial stem cell. Bioessays.

[3] Potten CS (1998). Stem cells in gastrointestinal epithelium: numbers, characteristics and death. Philos Trans R Soc Lond B Biol Sci.

[4] Gregorieff A, Stange DE, Kujala P, Begthel H, Van den Born M, Korving J, Peters PJ, Clevers H (2009). The ets-domain transcription factor Spdef promotes maturation of goblet and paneth cells in the intestinal epithelium. Gastroenterology.

[5] Gunawardene AR, Corfe BM, Staton CA (2011). Classification and functions of enteroendocrine cells of the lower gastrointestinal tract. International journal of experimental pathology.

[6] Sternini C, Anselmi L, Rozengurt E (2008). Enteroendocrine cells: a site of ‘taste’in gastrointestinal chemosensing. Current opinion in endocrinology, diabetes, and obesity.

[7] Spradling A, Drummond-Barbosa D, Kai T (2001). Stem cells find their niche. Nature.

[8] Crosnier C, Stamataki D, Lewis J (2006). Organizing cell renewal in the intestine: stem cells, signals and combinatorial control. Nat Rev Genet.

[9] Stappenbeck T (2010). The role of autophagy in Paneth cell differentiation and secretion. Mucosal immunology.

[10] Gerbe F, van Es JH, Makrini L, Brulin B, Mellitzer G, Robine S, Romagnolo B, Shroyer NF, Bourgaux J-F, Pignodel C (2011). Distinct ATOH1 and Neurog3 requirements define tuft cells as a new secretory cell type in the intestinal epithelium. The Journal of Cell Biology.

[11] Wright NA, Irwin M (1982). The kinetics of villus cell populations in the mouse small intestine. Cell proliferation.

[12] van der Flier LG, Clevers H (2009). Stem cells, self-renewal, and differentiation in the intestinal epithelium. Annual review of physiology.

[13] Carulli AJ, Samuelson LC, Schnell S (2014). Unraveling intestinal stem cell behavior with models of crypt dynamics. Integr Biol (Camb).

[14] Loeffler M, Stein R, Wichmann HE, Potten CS, Kaur P, Chwalinski S (1986). Intestinal cell proliferation. I. A comprehensive model of steady-state proliferation in the crypt. Cell and tissue kinetics.

[15] Potten CS, Loeffler M (1987). A comprehensive model of the crypts of the small intestine of the mouse provides insight into the mechanisms of cell migration and the proliferation hierarchy. Journal of Theoretical Biology.

[16] Meineke FA, Potten CS, Loeffler M (2001). Cell migration and organization in the intestinal crypt using a lattice-free model. Cell proliferation.

[17] van Leeuwen IM, Mirams GR, Walter A, Fletcher A, Murray P, Osborne J, Varma S, Young SJ, Cooper J, Doyle B (2009). An integrative computational model for intestinal tissue renewal. Cell proliferation.

[18] Fletcher AG, Breward CJ, Jonathan Chapman S (2012). Mathematical modeling of monoclonal conversion in the colonic crypt. J Theor Biol.

[19] Pin C, Watson AJ, Carding SR (2012). Modelling the spatio-temporal cell dynamics reveals novel insights on cell differentiation and proliferation in the small intestinal crypt. PloS one.

[20] Bravo R, Axelrod DE (2013). A calibrated agent-based computer model of stochastic cell dynamics in normal human colon crypts useful for in silico experiments. Theoretical Biology and Medical Modelling.

[21] Sklar E (2007). NetLogo, a Multi-agent Simulation Environment. Artificial Life.

[22] Buske P, Galle J, Barker N, Aust G, Clevers H, Loeffler M (2011). A Comprehensive Model of the Spatio-Temporal Stem Cell and Tissue Organisation in the Intestinal Crypt. PLOS Computational Biology.

[23] Buske P, Przybilla J, Loeffler M, Sachs N, Sato T, Clevers H, Galle J (2012). On the biomechanics of stem cell niche formation in the gut--modelling growing organoids. The FEBS journal.

[24] Wright NA, Alison M (1984). The Biology of Epithelial Cell Populations.

[25] Merzel J, Leblond CP (1969). Origin and renewal of goblet cells in the epithelium of the mouse small intestine. American Journal of Anatomy.

[26] Cheng H (1974). Origin, differentiation and renewal of the four main epithelial cell types in the mouse small intestine II. Mucous cells. American Journal of Anatomy.

[27] Noah TK, Kazanjian A, Whitsett J, Shroyer NF, Pointed Domain SAM, Factor ETS (2010). (SPDEF) regulates terminal differentiation and maturation of intestinal goblet cells. Experimental cell research.

[28] Shroyer NF, Wallis D, Venken KJ, Bellen HJ, Zoghbi HY (2005). Gfi1 functions downstream of Math1 to control intestinal secretory cell subtype allocation and differentiation. Genes & development.

[29] Garabedian EM, Roberts LJ, McNevin MS, Gordon JI (1997). Examining the role of Paneth cells in the small intestine by lineage ablation in transgenic mice. The Journal of biological chemistry.

[30] VanDussen KL, Carulli AJ, Keeley TM, Patel SR, Puthoff BJ, Magness ST, Tran IT, Maillard I, Siebel C, Kolterud A (2012). Notch signaling modulates proliferation and differentiation of intestinal crypt base columnar stem cells. Development (Cambridge, England).

[31] Johnston MD, Edwards CM, Bodmer WF, Maini PK, Chapman SJ (2007). Mathematical modeling of cell population dynamics in the colonic crypt and in colorectal cancer. Proc Natl Acad Sci U S A.

[32] Boyce WE, DiPrima RC. Elementary Differential Equations and Boundary Value Problems. 6th ed: New York: John Wiley & Sons, Inc.; 1997.

[33] Roth KA, Kim S, Gordon JI (1992). Immunocytochemical studies suggest two pathways for enteroendocrine cell differentiation in the colon. The American journal of physiology.

[34] Simons BD, Clevers H (2011). Stem cell self-renewal in intestinal crypt. Experimental cell research.

[35] Barker N, van de Wetering M, Clevers H (2008). The intestinal stem cell. Genes & development.

[36] Taylor JR. Introduction to Error Analysis: The Study of Uncertainties in Physical Measurements. 2nd ed. Sausalito: University Science Books; 1997.

[37] Barthel ER, Pierce JR, Goodhue CJ, Ford HR, Grikscheit TC, Upperman JS (2011). Availability of a pediatric trauma center in a disaster surge decreases triage time of the pediatric surge population: a population kinetics model. Theoretical Biology and Medical Modelling.

[38] Atherton RW, Schainker RB, Ducot ER (1975). On the statistical sensitivity analysis of models for chemical kinetics. AIChE J.

[39] Al-Nafussi AI, Wright NA (1982). Circadian rhythm in the rate of cellular proliferation and in the size of the functional compartment of mouse jejunal epithelium. Virchows Archiv B, Cell pathology including molecular pathology.

