# Supplementary material for: On the utility of a compartmental population kinetics model of intestinal epithelial stem cell proliferation and differentiation
Source: Theor Biol Med Model. 2017 Dec 19;14:25. doi: 10.1186/s12976-017-0071-8 (PMC5735948; doi:10.1186/s12976-017-0071-8)
Supplement: Supplementary file 4 — Supplemental Partial Derivatives (DOCX 23 kb) [file 12976_2017_71_MOESM4_ESM.docx]

This file contains the elements for the 9 x 19 matrix of partial derivatives of time-infinity values of each cell type with respect to each parameter. The subscript $t\to\infty$has been dropped from each cell population to make the notation less cumbersome. Note that ${CBC}_{0}$ is a constant, so all its partial derivatives vanish, and that the condition $k_{1}=\alpha$ (see main text) implies that for every cell population *X*, $\frac{\partial X}{\partial\alpha}=\frac{\partial X}{\partial k_{1}}$.

$\frac{\partial TAC}{\partial k_{1}}=\frac{{CBC}_{0}}{\omega_{d}}$

$\frac{\partial TAC}{\partial k_{2}}=\frac{\partial TAC}{\partial k_{3}}=-\frac{k_{1}}{\omega_{d}^{2}}{CBC}_{0}$

$\frac{\partial TAC}{\partial\beta}=\frac{k_{1}}{\omega_{d}^{2}}{CBC}_{0}$

$\frac{\partial TAC}{\partial k_{4-9}}=\frac{\partial TAC}{\partial\gamma}=\frac{\partial TAC}{\partial\delta}=\frac{\partial TAC}{\partial\zeta}=\frac{\partial TAC}{\partial\lambda_{i}}=0$

$\frac{\partial AP}{\partial k_{1}}=\frac{k_{2}}{\omega_{d}\omega_{e}}{CBC}_{0}$

$\frac{\partial AP}{\partial k_{2}}=\frac{k_{1}}{\omega_{d}\omega_{e}}{CBC}_{0}\left( 1-\frac{k_{2}}{\omega_{d}} \right)$

$\frac{\partial AP}{\partial k_{3}}=-\frac{k_{1}k_{2}}{\omega_{d}^{2}\omega_{e}}{CBC}_{0}$

$\frac{\partial AP}{\partial k_{5}}=-\frac{k_{1}k_{2}}{{\omega_{d}\omega}_{e}^{2}}{CBC}_{0}$

$\frac{\partial AP}{\partial\beta}=\frac{k_{1}k_{2}}{\omega_{d}^{2}\omega_{e}}{CBC}_{0}$

$\frac{\partial AP}{\partial\gamma}=\frac{k_{1}k_{2}}{{\omega_{d}\omega}_{e}^{2}}{CBC}_{0}$

$\frac{\partial AP}{\partial k_{4,6-9}}=\frac{\partial AP}{\partial\delta}=\frac{\partial AP}{\partial\zeta}=\frac{\partial AP}{\partial\lambda_{i}}=0$

$\frac{\partial EC}{\partial k_{1}}=\frac{k_{2}k_{5}}{\omega_{d}\omega_{e}\lambda_{1}}{CBC}_{0}$

$\frac{\partial EC}{\partial k_{2}}=\frac{k_{1}k_{5}}{\omega_{d}\omega_{e}\lambda_{1}}{CBC}_{0}\left( 1-\frac{k_{2}}{\omega_{d}} \right)$

$\frac{\partial EC}{\partial k_{3}}=-\frac{k_{1}k_{2}k_{5}}{\omega_{d}^{2}\omega_{e}\lambda_{1}}{CBC}_{0}$

$\frac{\partial EC}{\partial k_{5}}=\frac{k_{1}k_{2}}{\omega_{d}\omega_{e}\lambda_{1}}{CBC}_{0}\left( 1-\frac{k_{5}}{\omega_{e}} \right)$

$\frac{\partial EC}{\partial\beta}=\frac{k_{1}k_{2}k_{5}}{\omega_{d}^{2}\omega_{e}\lambda_{1}}{CBC}_{0}$

$\frac{\partial EC}{\partial\gamma}=\frac{k_{1}k_{2}k_{5}}{{\omega_{d}\omega}_{e}^{2}\lambda_{1}}{CBC}_{0}$

$\frac{\partial EC}{\partial\lambda_{1}}=-\frac{k_{1}k_{2}k_{5}}{{\omega_{d}\omega_{e}\lambda}_{1}^{2}}{CBC}_{0}$

$\frac{\partial EC}{\partial k_{4,6-9}}=\frac{\partial EC}{\partial\delta}=\frac{\partial EC}{\partial\zeta}=\frac{\partial EC}{\partial\lambda_{2-5}}=0$

$\frac{\partial SP}{\partial k_{1}}=\frac{k_{3}}{\omega_{d}\omega_{s}}{CBC}_{0}$

$\frac{\partial SP}{\partial k_{2}}=-\frac{k_{1}k_{3}}{\omega_{d}^{2}\omega_{s}}{CBC}_{0}$

$\frac{\partial SP}{\partial k_{3}}=\frac{k_{1}}{\omega_{d}\omega_{s}}{CBC}_{0}\left( 1-\frac{k_{3}}{\omega_{d}} \right)$

$\frac{\partial SP}{\partial k_{4}}=\frac{\partial SP}{\partial k_{6}}=\frac{\partial SP}{\partial k_{7}}=-\frac{k_{1}k_{3}}{\omega_{d}\omega_{s}^{2}}{CBC}_{0}$

$\frac{\partial SP}{\partial\beta}=\frac{k_{1}k_{3}}{\omega_{d}^{2}\omega_{s}}{CBC}_{0}$

$\frac{\partial SP}{\partial\delta}=\frac{k_{1}k_{3}}{\omega_{d}\omega_{s}^{2}}{CBC}_{0}$

$\frac{\partial SP}{\partial k_{5,8,9}}=\frac{\partial SP}{\partial\gamma}=\frac{\partial SP}{\partial\zeta}=\frac{\partial SP}{\partial\lambda_{i}}=0$

$\frac{\partial EEC}{\partial k_{1}}=\frac{k_{3}k_{6}}{\omega_{d}\omega_{s}\lambda_{2}}{CBC}_{0}$

$\frac{\partial EEC}{\partial k_{2}}=-\frac{k_{1}k_{3}k_{6}}{\omega_{d}^{2}\omega_{s}\lambda_{2}}{CBC}_{0}$

$\frac{\partial EEC}{\partial k_{3}}=\frac{k_{1}k_{6}}{\omega_{d}\omega_{s}\lambda_{2}}{CBC}_{0}\left( 1-\frac{k_{3}}{\omega_{d}} \right)$

$\frac{\partial EEC}{\partial k_{4}}=\frac{\partial EEC}{\partial k_{7}}=-\frac{k_{1}k_{3}k_{6}}{\omega_{d}\omega_{s}^{2}\lambda_{2}}{CBC}_{0}$

$\frac{\partial EEC}{\partial k_{6}}=\frac{k_{1}k_{3}}{\omega_{d}\omega_{s}\lambda_{2}}{CBC}_{0}\left( 1-\frac{k_{6}}{\omega_{s}} \right)$

$\frac{\partial EEC}{\partial\beta}=\frac{k_{1}k_{3}k_{6}}{\omega_{d}^{2}\omega_{s}\lambda_{2}}{CBC}_{0}$

$\frac{\partial EEC}{\partial\delta}=\frac{k_{1}k_{3}k_{6}}{\omega_{d}\omega_{s}^{2}\lambda_{2}}{CBC}_{0}$

$\frac{\partial EEC}{\partial\lambda_{2}}=-\frac{k_{1}k_{3}k_{6}}{\omega_{d}{\omega_{s}\lambda}_{2}^{2}}{CBC}_{0}$

$\frac{\partial EEC}{\partial k_{5,8,9}}=\frac{\partial EEC}{\partial\gamma}=\frac{\partial EEC}{\partial\zeta}=\frac{\partial EEC}{\partial\lambda_{1,3-5}}=0$

$\frac{\partial TC}{\partial k_{1}}=\frac{k_{3}k_{7}}{\omega_{d}\omega_{s}\lambda_{3}}{CBC}_{0}$

$\frac{\partial TC}{\partial k_{2}}=-\frac{k_{1}k_{3}k_{7}}{\omega_{d}^{2}\omega_{s}\lambda_{3}}{CBC}_{0}$

$\frac{\partial TC}{\partial k_{3}}=\frac{k_{1}k_{7}}{\omega_{d}\omega_{s}\lambda_{3}}{CBC}_{0}\left( 1-\frac{k_{3}}{\omega_{d}} \right)$

$\frac{\partial TC}{\partial k_{4}}=\frac{\partial TC}{\partial k_{6}}=-\frac{k_{1}k_{3}k_{7}}{{\omega_{d}\omega}_{s}^{2}\lambda_{3}}{CBC}_{0}$

$\frac{\partial TC}{\partial k_{7}}=\frac{k_{1}k_{3}}{\omega_{d}\omega_{s}\lambda_{3}}{CBC}_{0}\left( 1-\frac{k_{7}}{\omega_{s}} \right)$

$\frac{\partial TC}{\partial\beta}=\frac{k_{1}k_{3}k_{7}}{\omega_{d}^{2}\omega_{s}\lambda_{3}}{CBC}_{0}$

$\frac{\partial TC}{\partial\delta}=\frac{k_{1}k_{3}k_{7}}{\omega_{d}\omega_{s}^{2}\lambda_{3}}{CBC}_{0}$

$\frac{\partial EEC}{\partial\lambda_{3}}=-\frac{k_{1}k_{3}k_{7}}{\omega_{d}{\omega_{s}\lambda}_{3}^{2}}{CBC}_{0}$

$\frac{\partial TC}{\partial k_{5,8,9}}=\frac{\partial TC}{\partial\gamma}=\frac{\partial TC}{\partial\zeta}=\frac{\partial TC}{\partial\lambda_{1,2,4,5}}=0$

$\frac{\partial GPP}{\partial k_{1}}=\frac{k_{3}k_{4}}{\omega_{d}\omega_{m}\omega_{s}}{CBC}_{0}$

$\frac{\partial GPP}{\partial k_{2}}=-\frac{k_{1}k_{3}k_{4}}{{\omega_{d}^{2}\omega}_{m}\omega_{s}}{CBC}_{0}$

$\frac{\partial GPP}{\partial k_{3}}=\frac{k_{1}k_{4}}{\omega_{d}\omega_{m}\omega_{s}}{CBC}_{0}\left( 1-\frac{k_{3}}{\omega_{d}} \right)$

$\frac{\partial GPP}{\partial k_{4}}=\frac{k_{1}k_{3}}{\omega_{d}\omega_{m}\omega_{s}}{CBC}_{0}\left( 1-\frac{k_{4}}{\omega_{s}} \right)$

$\frac{\partial GPP}{\partial k_{6}}=\frac{\partial GPP}{\partial k_{7}}=-\frac{k_{1}k_{3}k_{4}}{\omega_{d}\omega_{m}\omega_{s}^{2}}{CBC}_{0}$

$\frac{\partial GPP}{\partial k_{8}}=\frac{\partial GPP}{\partial k_{9}}=-\frac{k_{1}k_{3}k_{4}}{\omega_{d}\omega_{m}^{2}\omega_{s}}{CBC}_{0}$

$\frac{\partial GPP}{\partial\beta}=\frac{k_{1}k_{3}k_{4}}{{\omega_{d}^{2}\omega}_{m}\omega_{s}}{CBC}_{0}$

$\frac{\partial GPP}{\partial\delta}=\frac{k_{1}k_{3}k_{4}}{\omega_{d}\omega_{m}\omega_{s}^{2}}{CBC}_{0}$

$\frac{\partial GPP}{\partial\zeta}=\frac{k_{1}k_{3}k_{4}}{\omega_{d}\omega_{m}^{2}\omega_{s}}{CBC}_{0}$

$\frac{\partial GPP}{{\partial k}_{5}}=\frac{\partial GPP}{\partial\gamma}=\frac{\partial GPP}{\partial\lambda_{i}}=0$

$\frac{\partial GC}{\partial k_{1}}=\frac{k_{3}k_{4}k_{8}}{\omega_{d}\omega_{m}\omega_{s}\lambda_{4}}{CBC}_{0}$

$\frac{\partial GC}{\partial k_{2}}=-\frac{k_{1}k_{3}k_{4}k_{8}}{\omega_{d}^{2}\omega_{m}\omega_{s}\lambda_{4}}{CBC}_{0}$

$\frac{\partial GC}{\partial k_{3}}=\frac{k_{1}k_{4}k_{8}}{\omega_{d}\omega_{m}\omega_{s}\lambda_{4}}{CBC}_{0}\left( 1-\frac{k_{3}}{\omega_{d}} \right)$

$\frac{\partial GC}{\partial k_{4}}=\frac{k_{1}k_{3}k_{8}}{\omega_{d}\omega_{m}\omega_{s}\lambda_{4}}{CBC}_{0}\left( 1-\frac{k_{4}}{\omega_{s}} \right)$

$\frac{\partial GC}{\partial k_{6}}=\frac{\partial GC}{\partial k_{7}}=-\frac{k_{1}k_{3}k_{4}k_{8}}{\omega_{d}\omega_{m}\omega_{s}^{2}\lambda_{4}}{CBC}_{0}$

$\frac{\partial GC}{\partial k_{8}}=\frac{k_{1}k_{3}k_{4}}{\omega_{d}\omega_{m}\omega_{s}\lambda_{4}}{CBC}_{0}\left( 1-\frac{k_{8}}{\omega_{m}} \right)$

$\frac{\partial GC}{\partial k_{9}}=-\frac{k_{1}k_{3}k_{4}k_{8}}{\omega_{d}\omega_{m}^{2}\omega_{s}\lambda_{4}}{CBC}_{0}$

$\frac{\partial GC}{\partial\beta}=\frac{k_{1}k_{3}k_{4}k_{8}}{\omega_{d}^{2}\omega_{m}\omega_{s}\lambda_{4}}{CBC}_{0}$

$\frac{\partial GC}{\partial\delta}=\frac{k_{1}k_{3}k_{4}k_{8}}{{\omega_{d}\omega}_{m}\omega_{s}^{2}\lambda_{4}}{CBC}_{0}$

$\frac{\partial GC}{\partial\zeta}=\frac{k_{1}k_{3}k_{4}k_{8}}{\omega_{d}\omega_{m}^{2}\omega_{s}\lambda_{4}}{CBC}_{0}$

$\frac{\partial GC}{\partial\lambda_{4}}=-\frac{k_{1}k_{3}k_{4}k_{8}}{\omega_{d}\omega_{m}{\omega_{s}\lambda}_{4}^{2}}{CBC}_{0}$

$\frac{\partial GC}{\partial k_{5}}=\frac{\partial GC}{\partial\gamma}=\frac{\partial GC}{\partial\lambda_{1-3,5}}=0$

$\frac{\partial PC}{\partial k_{1}}=\frac{k_{3}k_{4}k_{9}}{\omega_{d}\omega_{m}\omega_{s}\lambda_{5}}{CBC}_{0}$

$\frac{\partial PC}{\partial k_{2}}=-\frac{k_{1}k_{3}k_{4}k_{9}}{\omega_{d}^{2}\omega_{m}\omega_{s}\lambda_{5}}{CBC}_{0}$

$\frac{\partial PC}{\partial k_{3}}=\frac{k_{1}k_{4}k_{9}}{\omega_{d}\omega_{m}\omega_{s}\lambda_{5}}{CBC}_{0}\left( 1-\frac{k_{3}}{\omega_{d}} \right)$

$\frac{\partial PC}{\partial k_{4}}=\frac{k_{1}k_{3}k_{9}}{\omega_{d}\omega_{m}\omega_{s}\lambda_{5}}{CBC}_{0}\left( 1-\frac{k_{4}}{\omega_{s}} \right)$

$\frac{\partial PC}{\partial k_{6}}=\frac{\partial PC}{\partial k_{7}}=-\frac{k_{1}k_{3}k_{4}k_{9}}{\omega_{d}\omega_{m}\omega_{s}^{2}\lambda_{5}}{CBC}_{0}$

$\frac{\partial PC}{\partial k_{8}}=-\frac{k_{1}k_{3}k_{4}k_{9}}{\omega_{d}\omega_{m}^{2}\omega_{s}\lambda_{5}}{CBC}_{0}$

$\frac{\partial PC}{\partial k_{9}}=\frac{k_{1}k_{3}k_{4}}{\omega_{d}\omega_{m}\omega_{s}\lambda_{5}}{CBC}_{0}\left( 1-\frac{k_{9}}{\omega_{m}} \right)$

$\frac{\partial PC}{\partial\beta}=\frac{k_{1}k_{3}k_{4}k_{9}}{\omega_{d}^{2}\omega_{m}\omega_{s}\lambda_{5}}{CBC}_{0}$

$\frac{\partial PC}{\partial\delta}=\frac{k_{1}k_{3}k_{4}k_{9}}{\omega_{d}\omega_{m}\omega_{s}^{2}\lambda_{5}}{CBC}_{0}$

$\frac{\partial PC}{\partial\zeta}=\frac{k_{1}k_{3}k_{4}k_{9}}{\omega_{d}\omega_{m}^{2}\omega_{s}\lambda_{5}}{CBC}_{0}$

$\frac{\partial PC}{\partial k_{5}}=\frac{\partial PC}{\partial\gamma}=\frac{\partial PC}{\partial\lambda_{1-4}}=0$
