# Supplementary material for: On the utility of a compartmental population kinetics model of intestinal epithelial stem cell proliferation and differentiation
Source: Theor Biol Med Model. 2017 Dec 19;14:25. doi: 10.1186/s12976-017-0071-8 (PMC5735948; doi:10.1186/s12976-017-0071-8)
Supplement: Supplementary file 6 — Supplemental Tables. (DOCX 35 kb) [file 12976_2017_71_MOESM6_ESM.docx]

Table S1. Derivative Matrix ***S*** of model outputs with respect to parameters for baseline scenario.

|  | Parameter partial derivative | | | | | | | | | | | | | | | | | | |
| --- | --- | --- | --- | --- | --- | --- | --- | --- | --- | --- | --- | --- | --- | --- | --- | --- | --- | --- | --- |
| Output | $\frac{\partial}{\partial k_{1}}$ | $\frac{\partial}{\partial k_{2}}$ | $\frac{\partial}{\partial k_{3}}$ | $\frac{\partial}{\partial k_{4}}$ | $\frac{\partial}{\partial k_{5}}$ | $\frac{\partial}{\partial k_{6}}$ | $\frac{\partial}{\partial k_{7}}$ | $\frac{\partial}{\partial k_{8}}$ | $\frac{\partial}{\partial k_{9}}$ | $\frac{\partial}{\partial\alpha}$ | $\frac{\partial}{\partial\beta}$ | $\frac{\partial}{\partial\gamma}$ | $\frac{\partial}{\partial\delta}$ | $\frac{\partial}{\partial\zeta}$ | $\frac{\partial}{\partial\lambda_{1}}$ | $\frac{\partial}{\partial\lambda_{2}}$ | $\frac{\partial}{\partial\lambda_{3}}$ | $\frac{\partial}{\partial\lambda_{4}}$ | $\frac{\partial}{\partial\lambda_{5}}$ |
| ${TAC}_{t\to\infty}$ | 310 | -9610 | -9610 | 0 | 0 | 0 | 0 | 0 | 0 | 310 | 9610 | 0 | 0 | 0 | 0 | 0 | 0 | 0 | 0 |
| ${AP}_{t\to\infty}$ | 271 | -8240 | -8400 | 0 | -138 | 0 | 0 | 0 | 0 | 271 | 8400 | 138 | 0 | 0 | 0 | 0 | 0 | 0 | 0 |
| ${EC}_{t\to\infty}$ | 2844 | -86500 | -88200 | 0 | -684 | 0 | 0 | 0 | 0 | 2844 | 88200 | 1450 | 0 | 0 | -8040 | 0 | 0 | 0 | 0 |
| ${SP}_{t\to\infty}$ | 49.0 | -1519 | -796 | -114 | 0 | -114 | -114 | 0 | 0 | 49.0 | 1520 | 0 | 114 | 0 | 0 | 0 | 0 | 0 | 0 |
| ${EEC}_{t\to\infty}$ | 40.0 | -1240 | -650 | -93.3 | 0 | -18.9 | -93.3 | 0 | 0 | 40.0 | 1240 | 0 | 93.3 | 0 | 0 | -91.6 | 0 | 0 | 0 |
| ${TC}_{t\to\infty}$ | 16.0 | -496 | -260 | -37.3 | 0 | -37.3 | 74.9 | 0 | 0 | 16.0 | 496 | 0 | 37.3 | 0 | 0 | 0 | -36.6 | 0 | 0 |
| ${GPP}_{t\to\infty}$ | 40.0 | -1240 | -650 | -69.5 | 0 | -93.3 | -93.3 | -19.4 | -19.4 | 40.0 | 1240 | 0 | 93.3 | 19.4 | 0 | 0 | 0 | 0 | 0 |
| ${GC}_{t\to\infty}$ | 340 | -10540 | -5520 | -591 | 0 | -793 | -793 | -73.7 | -165 | 340 | 10500 | 0 | 793 | 165 | 0 | 0 | 0 | -778 | 0 |
| ${PC}_{t\to\infty}$ | 80.0 | -2480 | -1300 | -140 | 0 | -187 | -187 | -38.9 | 801 | 80.0 | 2480 | 0 | 187 | 38.9 | 0 | 0 | 0 | 0 | -1680 |

Table S2. Variance Matrix ***V*** of model outputs for baseline scenario ($V_{ij}=S_{ij}^{2}\sigma_{j}^{2}$).

|  | Parameter uncertainty | | | | | | | | | | | | | | | | | | |
| --- | --- | --- | --- | --- | --- | --- | --- | --- | --- | --- | --- | --- | --- | --- | --- | --- | --- | --- | --- |
| Output | $k_{1}$ | $k_{2}$ | $k_{3}$ | $k_{4}$ | $k_{5}$ | $k_{6}$ | $k_{7}$ | $k_{8}$ | $k_{9}$ | $\alpha$ | $\beta$ | $\gamma$ | $\delta$ | $\zeta$ | $\lambda_{1}$ | $\lambda_{2}$ | $\lambda_{3}$ | $\lambda_{4}$ | $\lambda_{5}$ |
| ${TAC}_{t\to\infty}$ | 6010 | 5.45x10^-3^ | 3.36x10^5^ | 0 | 0 | 0 | 0 | 0 | 0 | 6010 | 5.77x10^6^ | 0 | 0 | 0 | 0 | 0 | 0 | 0 | 0 |
| ${AP}_{t\to\infty}$ | 4590 | 4.01x10^-3^ | 2.56x10^5^ | 0 | 1.59 x10^-2^ | 0 | 0 | 0 | 0 | 4590 | 4.41x10^6^ | 1200 | 0 | 0 | 0 | 0 | 0 | 0 | 0 |
| ${EC}_{t\to\infty}$ | 5.06x10^5^ | 0.442 | 2.82x10^7^ | 0 | 0.390 | 0 | 0 | 0 | 0 | 5.06x10^5^ | 4.86x10^8^ | 1.31x10^5^ | 0 | 0 | 0.351 | 0 | 0 | 0 | 0 |
| ${SP}_{t\to\infty}$ | 150 | 1.36x10^-4^ | 2300 | 3.26x10^-5^ | 0 | 6.85 x10^-4^ | 133 | 0 | 0 | 150 | 1.44x10^5^ | 0 | 816 | 0 | 0 | 0 | 0 | 0 | 0 |
| ${EEC}_{t\to\infty}$ | 100 | 9.08x10^-5^ | 1530 | 2.17x10^-5^ | 0 | 1.87x10^-5^ | 88.5 | 0 | 0 | 100 | 9.61x10^4^ | 0 | 544 | 0 | 0 | 1.87x10^-5^ | 0 | 0 | 0 |
| ${TC}_{t\to\infty}$ | 16.0 | 1.45x10^-5^ | 245 | 3.47x10^-6^ | 0 | 7.31x10^-5^ | 57.0 | 0 | 0 | 16.0 | 1.54x10^4^ | 0 | 87 | 0 | 0 | 0 | 2.99x10^-6^ | 0 | 0 |
| ${GPP}_{t\to\infty}$ | 100 | 9.08x10^-5^ | 1530 | 1.20x10^-5^ | 0 | 4.57x10^-4^ | 88.5 | 2.78x10^-2^ | 0.755 | 100 | 9.61x10^4^ | 0 | 544 | 23.6 | 0 | 0 | 0 | 0 | 0 |
| ${GC}_{t\to\infty}$ | 7220 | 6.56x10^-3^ | 1.11x10^5^ | 8.70 x10^-4^ | 0 | 3.30x10^-2^ | 6400 | 0.398 | 54.5 | 7220 | 6.94x10^6^ | 0 | 3.93x10^4^ | 1710 | 0 | 0 | 0 | 1.35x10^-3^ | 0 |
| ${PC}_{t\to\infty}$ | 400 | 3.63x10^-4^ | 6140 | 4.82x10^-5^ | 0 | 1.83x10^-3^ | 354 | 0.111 | 1280 | 400 | 3.84x10^5^ | 0 | 2180 | 94.5 | 0 | 0 | 0 | 0 | 256 |

Table S3. Derivative Matrix ***S*** of model outputs with respect to parameters for fast scenario.

|  | Parameter partial derivative | | | | | | | | | | | | | | | | | | |
| --- | --- | --- | --- | --- | --- | --- | --- | --- | --- | --- | --- | --- | --- | --- | --- | --- | --- | --- | --- |
| Output | $\frac{\partial}{\partial k_{1}}$ | $\frac{\partial}{\partial k_{2}}$ | $\frac{\partial}{\partial k_{3}}$ | $\frac{\partial}{\partial k_{4}}$ | $\frac{\partial}{\partial k_{5}}$ | $\frac{\partial}{\partial k_{6}}$ | $\frac{\partial}{\partial k_{7}}$ | $\frac{\partial}{\partial k_{8}}$ | $\frac{\partial}{\partial k_{9}}$ | $\frac{\partial}{\partial\alpha}$ | $\frac{\partial}{\partial\beta}$ | $\frac{\partial}{\partial\gamma}$ | $\frac{\partial}{\partial\delta}$ | $\frac{\partial}{\partial\zeta}$ | $\frac{\partial}{\partial\lambda_{1}}$ | $\frac{\partial}{\partial\lambda_{2}}$ | $\frac{\partial}{\partial\lambda_{3}}$ | $\frac{\partial}{\partial\lambda_{4}}$ | $\frac{\partial}{\partial\lambda_{5}}$ |
| ${TAC}_{t\to\infty}$ | 12.9 | -400 | -400 | 0 | 0 | 0 | 0 | 0 | 0 | 12.9 | 400 | 0 | 0 | 0 | 0 | 0 | 0 | 0 | 0 |
| ${AP}_{t\to\infty}$ | 11.3 | -308 | -350 | 0 | -36.8 | 0 | 0 | 0 | 0 | 11.3 | 350 | 36.8 | 0 | 0 | 0 | 0 | 0 | 0 | 0 |
| ${EC}_{t\to\infty}$ | 118 | -3230 | -3670 | 0 | -195 | 0 | 0 | 0 | 0 | 118 | 3670 | 387 | 0 | 0 | -2010 | 0 | 0 | 0 | 0 |
| ${SP}_{t\to\infty}$ | 1.87 | -58.1 | 302 | -52.3 | 0 | -52.3 | -52.3 | 0 | 0 | 1.87 | 58.1 | 0 | 52.3 | 0 | 0 | 0 | 0 | 0 | 0 |
| ${EEC}_{t\to\infty}$ | 1.66 | -51.6 | 269 | -46.5 | 0 | 334 | -46.5 | 0 | 0 | 1.66 | 51.6 | 0 | 46.5 | 0 | 0 | -338 | 0 | 0 | 0 |
| ${TC}_{t\to\infty}$ | 0.666 | -20.6 | 107 | -18.6 | 0 | -18.6 | 286 | 0 | 0 | 0.666 | 20.6 | 0 | 18.6 | 0 | 0 | 0 | -108 | 0 | 0 |
| ${GPP}_{t\to\infty}$ | 1.66 | -51.6 | 269 | 3.61 | 0 | -46.5 | -46.5 | -44.5 | -44.5 | 1.66 | 51.6 | 0 | 46.5 | 44.5 | 0 | 0 | 0 | 0 | 0 |
| ${GC}_{t\to\infty}$ | 14.2 | -439 | 2280 | 30.7 | 0 | -395 | -395 | -122 | -378 | 14.2 | 439 | 0 | 395 | 378 | 0 | 0 | 0 | -2180 | 0 |
| ${PC}_{t\to\infty}$ | 3.33 | -103 | 537 | 7.22 | 0 | -93.0 | -93.0 | -89.0 | 746 | 3.33 | 103 | 0 | 93.0 | 89.0 | 0 | 0 | 0 | 0 | -1670 |

Table S4. Variance Matrix ***V*** of model outputs for fast scenario ($V_{ij}=S_{ij}^{2}\sigma_{j}^{2}$).

|  | Parameter uncertainty | | | | | | | | | | | | | | | | | | |
| --- | --- | --- | --- | --- | --- | --- | --- | --- | --- | --- | --- | --- | --- | --- | --- | --- | --- | --- | --- |
| Output | $k_{1}$ | $k_{2}$ | $k_{3}$ | $k_{4}$ | $k_{5}$ | $k_{6}$ | $k_{7}$ | $k_{8}$ | $k_{9}$ | $\alpha$ | $\beta$ | $\gamma$ | $\delta$ | $\zeta$ | $\lambda_{1}$ | $\lambda_{2}$ | $\lambda_{3}$ | $\lambda_{4}$ | $\lambda_{5}$ |
| ${TAC}_{t\to\infty}$ | 5.72x10^-3^ | 7.05x10^-3^ | 1880 | 0 | 0 | 0 | 0 | 0 | 0 | 5.72x10^-3^ | 5.15x10^-3^ | 0 | 0 | 0 | 0 | 0 | 0 | 0 | 0 |
| ${AP}_{t\to\infty}$ | 4.37x10^-3^ | 4.17x10^-3^ | 1430 | 0 | 2.38x10^-2^ | 0 | 0 | 0 | 0 | 4.37x10^-3^ | 3.94x10^-3^ | 6.28x10^-3^ | 0 | 0 | 0 | 0 | 0 | 0 | 0 |
| ${EC}_{t\to\infty}$ | 0.482 | 0.460 | 1.58x10^5^ | 0 | 0.671 | 0 | 0 | 0 | 0 | 0.482 | 0.435 | 0.693 | 0 | 0 | 0.699 | 0 | 0 | 0 | 0 |
| ${SP}_{t\to\infty}$ | 1.20x10^-4^ | 1.49x10^-4^ | 1070 | 3.17x10^-3^ | 0 | 6.33x10^-5^ | 3.78 | 0 | 0 | 1.20x10^-4^ | 1.09x10^-4^ | 0 | 4.64x10^-3^ | 0 | 0 | 0 | 0 | 0 | 0 |
| ${EEC}_{t\to\infty}$ | 9.51x10^-5^ | 1.17x10^-4^ | 846 | 2.51x10^-3^ | 0 | 2.59x10^-3^ | 2.99 | 0 | 0 | 9.51x10^-5^ | 8.57x10^-5^ | 0 | 3.67x10^-3^ | 0 | 0 | 2.86x10^-3^ | 0 | 0 | 0 |
| ${TC}_{t\to\infty}$ | 1.52x10^-5^ | 1.88x10^-5^ | 135 | 4.01x10^-4^ | 0 | 8.00x10^-6^ | 113 | 0 | 0 | 1.52x10^-5^ | 1.37x10^-5^ | 0 | 5.86x10^-4^ | 0 | 0 | 0 | 0.157 | 0 | 0 |
| ${GPP}_{t\to\infty}$ | 9.51x10^-5^ | 1.17x10^-4^ | 846 | 1.51x10^-5^ | 0 | 5.00x10^-5^ | 2.99 | 3.84x10^-2^ | 3.28 | 9.51x10^-5^ | 8.57x10^-5^ | 0 | 3.66x10^-3^ | 6.47x10^-3^ | 0 | 0 | 0 | 0 | 0 |
| ${GC}_{t\to\infty}$ | 6.87x10^-3^ | 8.48x10^-3^ | 61100 | 1.09x10^-3^ | 0 | 3.61x10^-3^ | 216 | 0.288 | 237 | 6.87x10^-3^ | 6.20x10^-3^ | 0 | 0.265 | 0.468 | 0 | 0 | 0 | 0.594 | 0 |
| ${PC}_{t\to\infty}$ | 3.81x10^-4^ | 4.69x10^-4^ | 3380 | 6.05x10^-5^ | 0 | 2.00x10^-4^ | 11.9 | 0.153 | 922 | 3.81x10^-4^ | 3.43x10^-4^ | 0 | 1.47x10^-2^ | 2.59x10^-2^ | 0 | 0 | 0 | 0 | 2.28 |
